# Supplementary material for: Mechanisms governing the pioneering and redistribution capabilities of the non-classical pioneer PU.1
Source: Nat Commun. 2020 Jan 21;11:402. doi: 10.1038/s41467-019-13960-2 (PMC6972792; doi:10.1038/s41467-019-13960-2)
Supplement: Supplementary file 7 — Source data [file 41467_2019_13960_MOESM7_ESM.zip › Source_Data/FigureS5/FigureS5A_MotifsCommonPeaks/homerResults/motif12.similar.html]

motif12

## Information for motif12

C
T
A
G
T
C
A
G
G
A
C
T
A
T
C
G
G
T
C
A
T
A
G
C
C
G
A
T
T
G
A
C
C
T
G
A
A
G
C
T
  
Reverse Opposite:  

T
C
G
A
A
G
C
T
A
C
T
G
G
C
T
A
A
T
C
G
C
A
G
T
A
T
G
C
C
T
G
A
A
G
T
C
G
A
T
C
  

|  |  |
| --- | --- |
| p-value: | 1e-124 |
| log p-value: | -2.858e+02 |
| Information Content per bp: | 1.736 |
| Number of Target Sequences with motif | 787.0 |
| Percentage of Target Sequences with motif | 8.86% |
| Number of Background Sequences with motif | 1070.0 |
| Percentage of Background Sequences with motif | 2.82% |
| Average Position of motif in Targets | 97.6 +/- 53.4bp |
| Average Position of motif in Background | 99.9 +/- 61.3bp |
| Strand Bias (log2 ratio + to - strand density) | 0.0 |
| Multiplicity (# of sites on avg that occur together) | 1.04 |
| Motif File: | file (matrix) reverse opposite |

### Similar de novo motifs found

|  |  |  |  |  |  |  |  |
| --- | --- | --- | --- | --- | --- | --- | --- |
| Rank | Match Score | Redundant Motif | P-value | log P-value | % of Targets | % of Background | Motif file |
| 1 | 0.957 | T C A G C T G A C A G T T G C A C G A T T C A G T G C A T A G C C G A T G T A C G C T A | 1e-119 | -275.714321 | 9.32% | 3.16% | motif file (matrix) |
| 2 | 0.983 | T A C G C G T A T A C G A C G T A C T G C G T A A T G C A C G T A T G C C G T A A G T C G C T A | 1e-113 | -261.506025 | 5.35% | 1.15% | motif file (matrix) |
| 3 | 0.982 | G A C T G T A C C G T A T A C G A C G T A C T G C G T A A T G C A C G T A G T C C G T A A T G C G C A T | 1e-111 | -256.712839 | 5.53% | 1.25% | motif file (matrix) |
| 4 | 0.968 | C G A T A C T G G T C A T A C G A C G T T G A C C T G A A G C T | 1e-110 | -254.066433 | 7.51% | 2.29% | motif file (matrix) |
| 5 | 0.978 | C G T A T C G A A C G T A C T G C G T A A T C G A C G T G T A C C G T A | 1e-107 | -246.883544 | 7.89% | 2.56% | motif file (matrix) |
| 6 | 0.921 | A C G T A C T G C G T A A C T G A C G T A G T C C G T A | 1e-103 | -238.771509 | 8.27% | 2.85% | motif file (matrix) |
